# Allergen Immunotherapy and 36‐Month Allergic Respiratory Disease Trajectories: A Target‐Trial

**DOI:** 10.1002/iid3.70569

**Published:** 2026-10-26

**Authors:** Víctor Gonzalez‐Uribe, Sebastian Moreno‐Castro, Viviana Cardoso‐Perez, Diego A Mares‐Mateos, Mariel Paez‐Reyes, Jimena Prieto‐Gomez, Ricardo Martinez‐Tenopala, Paola de Baro Alvarez

**Affiliations:** ^1^ AlergiaMx Mexico City Mexico; ^2^ Facultad Mexicana de Medicina Universidad La Salle México Mexico City Mexico

**Keywords:** allergen immunotherapy, allergic asthma, disease progression, remission, target‐trial

## Abstract

**Background:**

Allergen immunotherapy (AIT) may modify allergic respiratory disease, but its association with progression and remission in routine practice remains uncertain.

**Materials and Methods:**

We emulated a target trial in a single‐center retrospective cohort at AlergiaMx, Mexico City, using data from 2020 to 2025. We compared sublingual allergen immunotherapy initiation within a 14‐day grace period with non‐initiation among AIT‐eligible patients. Follow‐up extended up to 36 months, and propensity‐score overlap weighting addressed baseline confounding.

**Results:**

Among 2,713 patients, 1,971 initiated AIT and 742 did not. Weighted disease worsening was 46.3% versus 63.6% (relative risk [RR] 0.73, 95% confidence interval [CI] 0.65–0.82), and sustained progression was 8.4% versus 17.0% (RR 0.49, 95% CI 0.35–0.69). At 36 months, clinical remission was 47.0% versus 18.3% (RR 2.57, 95% CI 1.93–3.43), and treatment‐associated remission was 31.7% versus 5.8% (RR 5.44, 95% CI 3.23–9.16). Longitudinal differences favored AIT for asthma control, quality of life, and fractional exhaled nitric oxide.

**Discussion:**

AIT initiation was associated with less worsening, greater remission, and favorable clinical and inflammatory trajectories over 36 months. These observational findings do not establish a causal disease‐modifying effect; residual confounding and differential follow‐up remain possible.

## Graphical abstract

Among 2,713 patients with allergic respiratory disease eligible for allergen immunotherapy (AIT), target‐trial compared 1,971 AIT initiators with 742 eligible non‐initiators over 36 months. AIT initiation was associated with less clinically meaningful worsening (46.3% vs 63.6%; RR 0.73), more clinical remission (47.0% vs 18.3%; RR 2.57), more treatment‐associated remission (31.7% vs 5.8%; RR 5.44), and a more favorable fractional exhaled nitric oxide trajectory. These findings support prospective evaluation of AIT as a modifier of allergic respiratory disease trajectories.

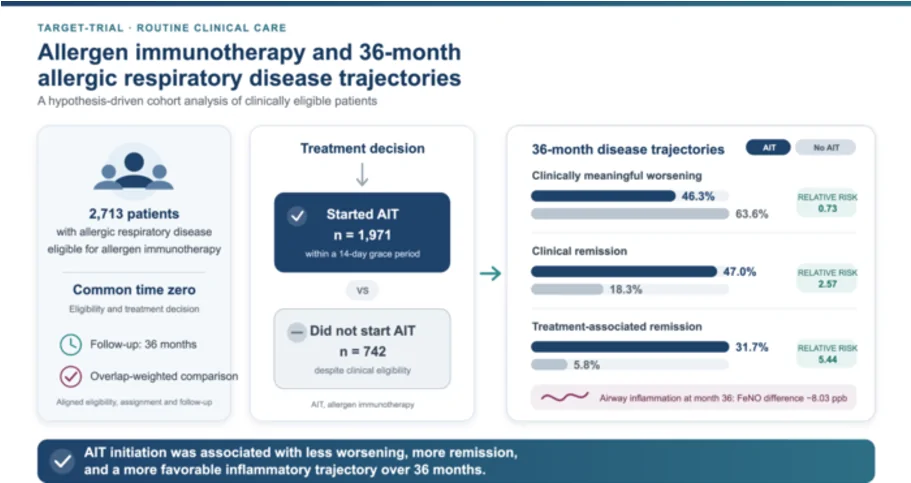

**Abbreviations**
ACQ: Asthma Control Questionnaire
ACT: Asthma Control Test
AIT: Allergen immunotherapy
CI: Confidence interval
FeNO: Fractional exhaled nitric oxide
GINA: Global Initiative for Asthma
IPCW: Inverse probability‐of‐censoring weighting
IQR: Interquartile range
OCS: Oral corticosteroid
OW: Overlap weighting
RR: Relative risk
SLIT: Sublingual immunotherapy
SMD: Standardized mean difference.

## 1 Introduction

Allergic respiratory diseases follow heterogeneous courses. Patients with allergic rhinitis and asthma may experience recurrent exacerbations, increasing treatment requirements, persistent loss of control, and extension of disease across organs; others achieve sustained control, treatment de‐escalation, or remission [1, 2]. Assessing isolated symptoms or short‐term physiological changes therefore provides an incomplete representation of the longitudinal disease course.

Allergen immunotherapy (AIT) is distinct from symptom‐directed pharmacotherapy because it targets the allergen‐specific immune response. Repeated administration of clinically relevant allergens promotes regulatory T‐ and B‐cell responses, changes allergen‐specific antibody profiles, reduces effector‐cell reactivity, and attenuates type 2 inflammation [3, 4, 5, 6]. Clinical benefits may persist beyond active treatment, supporting the concept of AIT as a potentially disease‐modifying immune therapy.

Disease modification should alter the probability of subsequent disease states rather than only improve one endpoint. In asthma, remission frameworks integrate disease control, exacerbation burden, and treatment requirements [7, 8, 9]. Whether AIT shifts the broader trajectory of allergic respiratory disease toward less progression, more remission, and reduced treatment intensity remains insufficiently characterized in routine practice.

Real‐world comparisons of patients who initiate AIT with those who remain untreated are vulnerable to confounding by indication, differential follow‐up, and misalignment of eligibility, treatment assignment, and time zero. Target‐trial emulation explicitly specifies the hypothetical trial an observational analysis seeks to approximate and can reduce design‐related bias [10, 11, 12]. We therefore compared AIT initiation with eligible non‐initiation over 36 months. We hypothesized that AIT would be associated with less clinically meaningful disease worsening, greater remission and treatment de‐escalation, and progressively favorable clinical and inflammatory trajectories.

## 2 Materials and Methods

### 2.1 Study Design and Target‐Trial Framework

We conducted a single‐center retrospective observational cohort study at AlergiaMx, a specialized allergy and clinical immunology center in Mexico City, Mexico, emulating a target trial comparing AIT initiation with eligible non‐initiation. The study period encompassed patient inclusion and follow‐up between 2020 and 2025. All included patients had index dates allowing a potential 36‐month follow‐up by study closure; actual follow‐up extended to month 36 or the last available assessment. Eligibility, strategies, assignment, time zero, follow‐up, outcomes, estimand, and analysis were prespecified (Table S1; Figure S1). Eligibility, treatment decision, and follow‐up start were aligned to reduce immortal‐time and prevalent‐user biases. The primary strategy approximated an intention‐to‐treat treatment‐policy estimand [10, 11, 12]. Reporting followed the STROBE framework with explicit specification of target‐trial components.

### 2.2 Study Population

The source population comprised patients with physician‐diagnosed allergic respiratory disease evaluated in routine allergy practice and considered for allergen‐specific treatment. Eligible participants had allergic rhinitis, asthma, or both; clinically relevant allergen sensitization; and sufficient baseline information to establish AIT eligibility and treatment strategy. Patients with allergic rhinitis without asthma were eligible because incident asthma was a prespecified manifestation of progression. Untreated comparators were restricted to patients clinically eligible for AIT.

### 2.3 Treatment Strategies and Time Zero

Time zero was the baseline clinical assessment at which AIT eligibility and treatment strategy were established, identically for both groups. AIT initiators began immunotherapy within 14 days (median 7 days, interquartile range [IQR] 3–11; range 0–14); untreated comparators did not initiate during this period. The grace period accommodated routine clinical logistics. Baseline covariates were restricted to information available at or before time zero. Documented reasons for non‐initiation included patient preference, preference for pharmacotherapy, cost or access barriers, adherence or logistical considerations, and deferred decisions.

AIT was administered by the sublingual route, with a planned treatment duration of 36 months. Formulations included house dust mites, Cupressus spp., Fraxinus excelsior, Quercus spp., Cynodon dactylon, and other aeroallergens. Adherence and treatment continuity were monitored using patient‐held written dosing calendars or administration logs together with center records of preparation of new immunotherapy vials and their delivery dates. These sources were used to verify reported dosing and treatment continuity for individual patients. The planned treatment duration was distinct from the availability of follow‐up assessments and did not imply that every participant completed 36 months of treatment.

### 2.4 Baseline Characteristics

Prespecified covariates included age, sex, pediatric status, allergic respiratory diagnosis, disease severity, sensitization burden, single‐ versus multiple‐group sensitization, blood eosinophils, total immunoglobulin E, FeNO, disease‐specific quality of life, previous exacerbations, and previous oral corticosteroid (OCS) bursts. Covariates for confounding control were selected on clinical grounds before outcome evaluation. Sensitization burden was counted by allergen group: positivity to several tree pollens contributed one group. Thus, group counts differ from the frequencies of individual aeroallergens listed in Table S9.

### 2.5 Outcomes

The primary outcome was clinically meaningful disease worsening during 36 months. In baseline asthma, worsening was defined by at least one asthma exacerbation; sustained escalation of at least one GINA step at two or more consecutive assessments; persistent deterioration in control (ACT decrease at least 3 points and/or ACQ increase at least 0.5 points at two or more consecutive assessments); or a sustained increase in systemic corticosteroid burden (at least one additional OCS burst relative to the pre‐index year during at least two annual intervals). In allergic rhinitis without baseline asthma, incident physician‐diagnosed asthma constituted worsening. Emergency visits and hospitalizations were analyzed separately and not counted as additional independent components of cumulative burden.

### 2.6 Sustained Progression, Remission, and Longitudinal Outcomes

Sustained disease progression excluded isolated exacerbations and was based on sustained treatment escalation, persistent loss of control, sustained increases in OCS burden, or incident asthma. Among patients with baseline asthma evaluable at month 36, clinical remission required controlled disease at month 36 plus no exacerbations or OCS bursts during months 24‐36. Controlled disease required ACT at least 20 and ACQ at most 0.75 when both were available; when only one validated instrument was available, its prespecified threshold was applied. Treatment‐associated remission additionally required at least one GINA‐step reduction or maintenance at the minimum clinically appropriate intensity. GINA step‐down was evaluated separately. Repeated outcomes included ACT, ACQ, disease‐specific quality of life, and FeNO (Table S2). These remission definitions describe an observed clinical state at month 36 and do not establish cure or remission after AIT discontinuation.

### 2.7 Propensity‐Score Estimation and Overlap Weighting

Baseline confounding was addressed with propensity‐score overlap weighting. The model included age, sex, diagnosis, severity, sensitized allergen‐group count, blood eosinophils, total immunoglobulin E, FeNO, baseline quality of life, exacerbations during the previous 12 months, and OCS bursts during the previous 12 months. AIT initiators received weights 1‐e(X), and untreated comparators received e(X), where e(X) was the estimated probability of initiation. Propensity‐score overlap was examined, and balance was assessed using absolute SMDs, with SMD below 0.10 considered adequate.

### 2.8 Statistical Analysis

Binary outcomes were analyzed using overlap‐weighted modified Poisson regression with robust standard errors to estimate relative risks and 95% CIs; weighted risks were also reported. Count outcomes were evaluated with overlap‐weighted negative‐binomial regression with robust standard errors. Repeated continuous outcomes were analyzed using overlap‐weighted longitudinal models incorporating baseline outcome values and follow‐up time; adjusted between‐group differences and 95% CIs were estimated at prespecified visits. All tests were two‐sided, and effect estimates were interpreted with their precision.

### 2.9 Missing Data, Sensitivity, and Subgroup Analyses

Follow‐up availability was evaluated at 2, 6, 12, 18, 24, and 36 months. Differential censoring was examined with stabilized inverse probability‐of‐censoring weights combined with overlap weights. Sensitivity analyses used a restrictive sustained‐progression endpoint, an expanded propensity model, stabilized inverse probability of treatment weighting truncated at the 1st and 99th percentiles, common‐support restriction, baseline‐asthma restriction, and restriction to participants retained through month 36. Exploratory heterogeneity was assessed by age, sex, baseline severity, sensitization status and burden, and diagnostic phenotype using treatment‐by‐subgroup interaction terms.

## 3 Results

### 3.1 Study Population and Target‐Trial Emulation

A total of 2,835 patients were identified, and 2,713 met the prespecified eligibility criteria (Figure 1). Among eligible participants, 1,971 initiated AIT and 742 formed the AIT‐eligible untreated comparator group. At baseline, 2,285 participants had physician‐diagnosed asthma (AIT, *n* = 1,664; untreated, *n* = 621), and 428 had allergic rhinitis without asthma. Propensity‐score overlap weighting improved covariate balance, with all post‐weighting absolute standardized mean differences (SMDs) below 0.10 (Table S3; Figure 2). At month 36, 1,712 of 2,713 participants (63.1%) remained evaluable; follow‐up availability was 65.2% among AIT initiators and 57.4% among untreated participants (Table S5).

**Figure 1:**
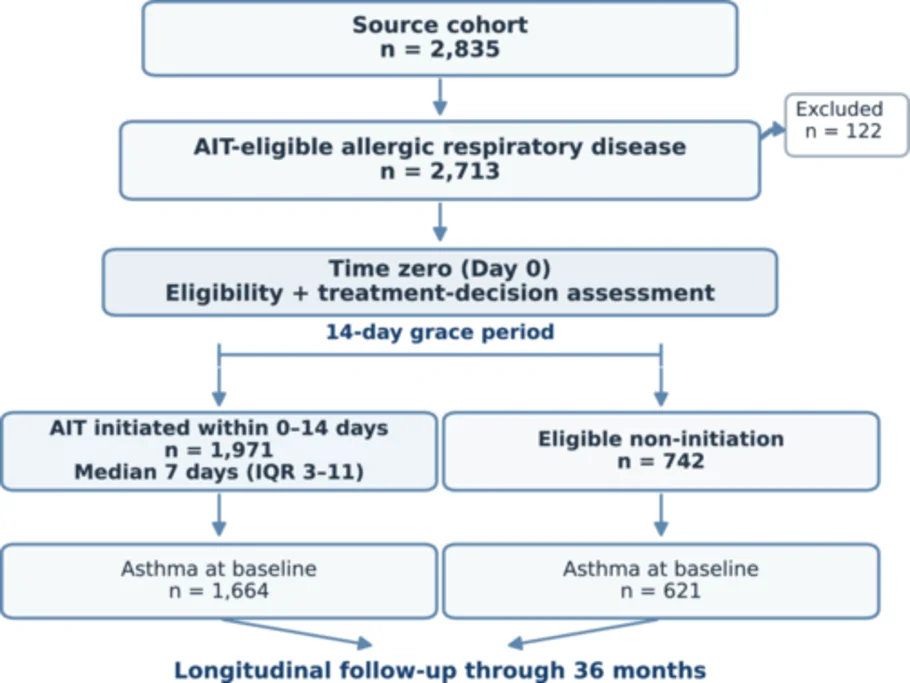
Cohort selection and target‐trial emulation. The source cohort included 2,835 patients; 2,713 met eligibility criteria, of whom 1,971 initiated allergen immunotherapy (AIT) within the 14‐day grace period and 742 remained untreated despite eligibility. Time zero was the common AIT‐eligibility and treatment‐decision assessment. AIT began at a median of 7 days (interquartile range 3–11; range 0–14), and follow‐up continued for up to 36 months or until the last available assessment.

**Figure 2:**
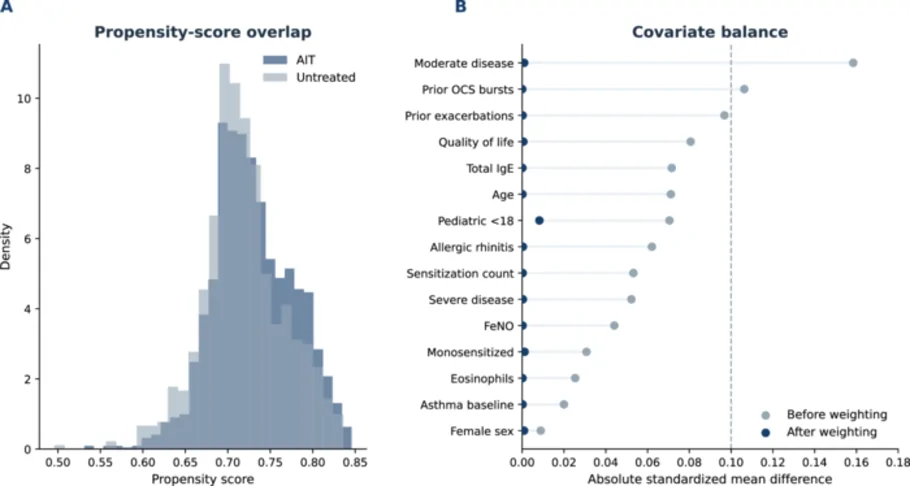
Propensity‐score overlap and covariate balance. The analysis included 2,713 eligible participants (AIT, *n* = 1,971; untreated, *n* = 742). (A) Distribution of estimated propensity scores. (B) Absolute standardized mean differences before and after overlap weighting; the dashed line marks the prespecified 0.10 balance threshold. Propensity scores were estimated using the prespecified baseline covariates described in Materials and methods. Balance was assessed with standardized differences rather than significance testing. Sensitization count and mono‐ versus polysensitization refer to allergen groups; several positive tree pollens count as one group.

### 3.2 Sensitization and SLIT Characteristics

Sensitization to the listed aeroallergens is summarized in Table S9. Among participants sensitized to a single allergen group, 23% were sensitized to house dust mites, 45% to trees, and 32% to other allergens. Among SLIT‐treated patients (*n* = 1,971), formulations included house dust mites in 65.6%, Cupressus spp. in 62.3%, Fraxinus excelsior in 48.7%, Quercus spp. in 33.7%, Cynodon dactylon in 24.0%, and other aeroallergens in 52.6% (Table S10). Treatment categories were not mutually exclusive. These formulation frequencies were reported separately from detected sensitization.

### 3.3 AIT Initiation and Clinically Meaningful Disease Worsening

During 36 months, clinically meaningful disease worsening occurred in weighted 46.3% of AIT initiators and 63.6% of untreated patients, corresponding to a 27% lower relative risk after AIT initiation (RR 0.73, 95% CI 0.65–0.82) (Table 1; Figure 3A). Among patients with asthma at baseline, AIT initiation was associated with fewer exacerbations (53.0% vs 71.9%), emergency department visits (15.0% vs 24.3%), hospitalizations (4.7% vs 8.1%), and sustained increases in oral corticosteroid burden (7.0% vs 15.3%) (Table S4). After isolated exacerbations were excluded, sustained disease progression remained less frequent with AIT (8.4% vs 17.0%; RR 0.49, 95% CI 0.35–0.69). Overall, 53.7% of AIT initiators remained free of any worsening component versus 36.4% of untreated patients; at least two independent worsening components occurred in 6.5% versus 13.7%, respectively.

**Table 1:** Primary and major secondary outcomes.

Outcome	AIT	Untreated	Effect estimate (95% CI)
Clinically meaningful disease worsening	46.3%	63.6%	RR 0.73 (0.65–0.82)
Sustained disease progression	8.4%	17.0%	RR 0.49 (0.35–0.69)
Clinical remission at 36 months	47.0%	18.3%	RR 2.57 (1.93–3.43)
Treatment‐associated remission	31.7%	5.8%	RR 5.44 (3.23–9.16)
GINA step‐down at 36 months	—	—	RR 4.20 (2.76–6.39)

*Note:* Values are overlap‐weighted risks. Relative risks and 95% confidence intervals were estimated using overlap‐weighted modified Poisson regression with robust standard errors.

Abbreviations: AIT, allergen immunotherapy; CI, confidence interval; RR, relative risk.

**Figure 3:**
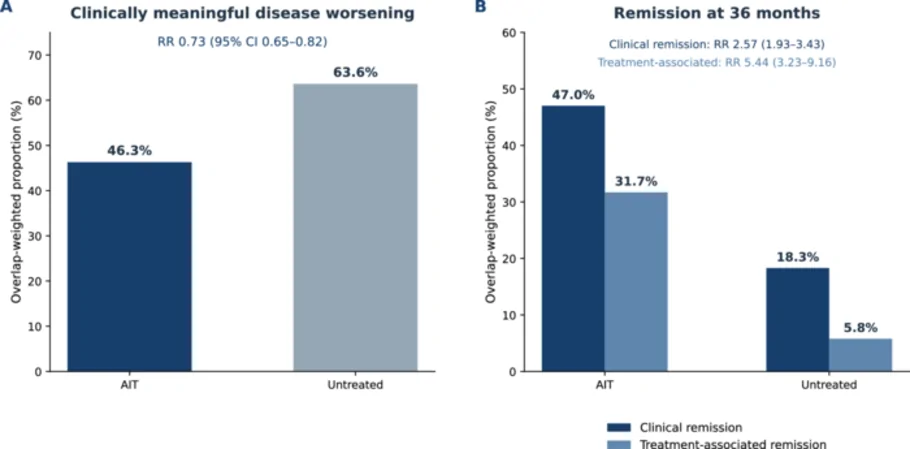
Disease worsening and remission. (A) Overlap‐weighted proportion with clinically meaningful disease worsening through 36 months in the full eligible cohort (AIT, *n* = 1,971; untreated, *n* = 742). (B) Overlap‐weighted clinical and treatment‐associated remission among patients with baseline asthma evaluable at month 36. Across the full cohort, 1,712/2,713 participants remained evaluable at month 36; group‐specific retention is reported in Table S5. Relative risks and 95% confidence intervals were estimated using overlap‐weighted modified Poisson regression with robust standard errors.

### 3.4 Remission and Treatment De‐Escalation

Among patients with asthma evaluable at 36 months, clinical remission was achieved by 47.0% of AIT initiators and 18.3% of untreated patients (RR 2.57, 95% CI 1.93–3.43) (Table 1; Figure 3B). Treatment‐associated remission occurred in 31.7% versus 5.8% (RR 5.44, 95% CI 3.23–9.16). Consistently, GINA step‐down at 36 months was more frequent after AIT initiation (RR 4.20, 95% CI 2.76–6.39).

### 3.5 Longitudinal Disease Control, Quality of Life, and Airway Inflammation

Differences between treatment strategies increased over follow‐up. At 12, 24, and 36 months, adjusted between‐group Asthma Control Test (ACT) differences were +1.34 (95% CI 1.19–1.48), +1.91 (1.74–2.08), and +2.12 (1.93–2.31) points. Corresponding Asthma Control Questionnaire (ACQ) differences were −0.28 (−0.30 to −0.25), −0.40 (−0.43 to −0.37), and −0.48 (−0.51 to −0.44). Quality‐of‐life differences were +0.40 (0.36–0.43), +0.60 (0.56–0.64), and +0.69 (0.64–0.74), while fractional exhaled nitric oxide (FeNO) differences were −4.93 ppb (−5.44 to −4.42), −6.92 ppb (−7.58 to −6.26), and −8.03 ppb (−8.73 to −7.32) (Figure 4; Table S8).

**Figure 4:**
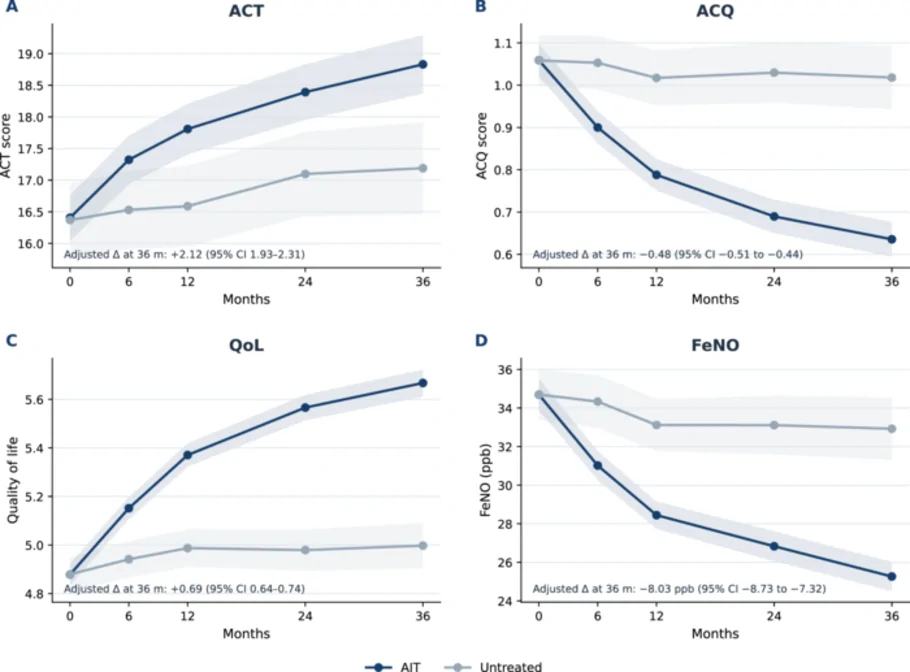
Longitudinal disease‐control and inflammatory trajectories through 36 months. Overlap‐weighted observed trajectories are shown for (A) Asthma Control Test, (B) Asthma Control Questionnaire, (C) disease‐specific quality of life, and (D) fractional exhaled nitric oxide. The parent cohort comprised 2,713 eligible participants (AIT, *n* = 1,971; untreated, *n* = 742); outcome‐specific models used available measurements at each visit, with follow‐up availability reported in Table S5. Shaded bands are 95% confidence intervals around weighted observed means. Annotations report adjusted between‐group differences at month 36 from overlap‐weighted longitudinal models incorporating baseline outcome and follow‐up time.

### 3.6 Robustness and Heterogeneity

The primary association was unchanged with an expanded propensity model, stabilized inverse probability of treatment weighting, common‐support restriction, restriction to baseline asthma, and analysis of participants retained through 36 months (RR range, 0.72–0.73) (Table S6). Inverse probability‐of‐censoring‐weighted remission estimates were similarly stable (Table S5). Associations were broadly consistent across age, sex, sensitization status and burden, and diagnostic phenotype. Possible attenuation with increasing baseline severity was observed (p for interaction=0.049) and was considered exploratory (Table S7).

## 4 Discussion

In this retrospective observational cohort using target‐trial emulation and up to 36 months of follow‐up in 2,713 patients with allergic respiratory disease, AIT initiation was associated with a more favorable disease trajectory than eligible non‐initiation. AIT initiators had less clinically meaningful disease worsening, more clinical and treatment‐associated remission, greater treatment de‐escalation, and progressively favorable changes in asthma control, quality of life, and FeNO. The findings remained stable across alternative outcome definitions, propensity‐score specifications, weighting strategies, common‐support restriction, baseline‐asthma restriction, complete follow‐up analysis, and censoring adjustment.

The principal finding was an association of AIT initiation with both less worsening and more remission. Worsening occurred in 46.3% of AIT initiators and 63.6% of untreated patients, whereas 36‐month clinical remission occurred in 47.0% and 18.3%, respectively. Treatment‐associated remission occurred in 31.7% versus 5.8%. A disease‐modifying intervention would be expected to reduce adverse future states while increasing sustained control, remission, and treatment reduction [7, 8, 9]. The concordant reduction in worsening and increase in remission provides a broader description of the observed clinical trajectories than either outcome alone, without establishing a causal disease‐modifying effect.

The primary composite captured multiple manifestations of deterioration. Although exacerbations contributed substantially, AIT was also associated with fewer emergency visits, hospitalizations, and sustained increases in systemic corticosteroid burden. Sustained progression remained approximately half as frequent after isolated exacerbations were removed. These findings extend database‐derived evidence of reduced medication use, exacerbations, and asthma progression after AIT by evaluating progression and remission as opposing dimensions of a longitudinal trajectory [13, 14, 15, 16].

Our findings complement long‐term real‐world evidence on AIT. In REACT, a large matched claims‐based cohort, AIT was associated with reduced rhinitis and asthma medication use, greater asthma treatment step‐down, and fewer severe exacerbations during follow‐up of up to 9 years [13]. Its predefined paediatric analysis also reported lower asthma medication use, severe exacerbations, and new oral corticosteroid prescriptions [16]; these findings arise from the same study programme rather than an independent replication. The TARGET prescription‐database study reported lower rhinitis and asthma medication requirements and reduced initiation of asthma medication after pollen allergoid subcutaneous immunotherapy, with at least 2 years of post‐treatment observation [17]. Prescription‐based outcomes provide evidence of treatment burden but are not equivalent to clinically adjudicated disease progression or remission.

Evidence of sustained benefit also requires attention to the outcome and treatment phase. In the randomized GAP trial, grass SLIT reduced asthma symptoms or medication use, including during the 2‐year post‐treatment period, but did not significantly alter the primary endpoint of time to asthma onset [18]. Against this background, our cohort adds a joint assessment of clinically defined worsening and month‐36 remission alongside treatment intensity, asthma control, quality of life, and FeNO. This broader clinical characterization complements database outcomes, but the observational design and absence of a defined post‐discontinuation evaluation prevent conclusions about causal or sustained off‐treatment disease modification.

The progressive separation in ACT, ACQ, quality of life, and FeNO adds biological coherence. FeNO is an accessible marker of type 2 airway inflammation, and its trajectory paralleled patient‐centered and treatment‐intensity outcomes. AIT induces allergen‐specific tolerance through regulatory lymphocyte responses, altered allergen‐specific antibody profiles, and reduced effector‐cell responsiveness [4, 5, 6]. The observed temporal pattern is compatible with, but does not establish, progressive immunomodulation. Importantly, greater GINA step‐down and treatment‐associated remission suggest that favorable disease states were not achieved solely through escalating background pharmacotherapy.

Methodologically, explicit alignment of eligibility, treatment decision, time zero, follow‐up, outcomes, and analysis was intended to reduce immortal‐time and prevalent‐user biases. Restricting comparators to clinically AIT‐eligible patients yielded a more relevant counterfactual than an unrestricted non‐AIT group, and overlap weighting achieved excellent measured balance [10, 11, 12]. These features strengthen interpretation while preserving the heterogeneity of routine clinical practice.

In this retrospective observational cohort, AIT initiation was associated with less clinically meaningful worsening, greater month‐36 remission, and more favorable clinical and inflammatory trajectories. These findings are compatible with a potential disease‐modifying role of AIT but do not establish causality. Target‐trial emulation and weighting address selected design and measured‐confounding concerns; residual confounding and differential follow‐up remain possible. The present outcomes do not demonstrate cure, off‐treatment remission, or persistence of benefit after AIT discontinuation. Prospective studies with standardized exposure characterization and post‐treatment follow‐up are needed to evaluate these questions.

## 5 Data Limitations and Perspectives

Residual and unmeasured confounding remain possible because motivation, adherence behavior, socioeconomic circumstances, physician preference, access to specialized care, and unmeasured disease complexity may influence both AIT initiation and outcomes. Estimates should not be interpreted as randomized treatment effects. The primary composite included phenotype‐dependent components, although individual components and a restrictive sustained‐progression analysis were concordant.

Differential loss to follow‐up is an important limitation. At month 36, follow‐up availability was 65.2% among AIT initiators and 57.4% among untreated participants. Continued attendance may be related to treatment engagement, access to care, perceived improvement, or persistent symptoms; consequently, the direction and magnitude of selection bias cannot be determined from the observed retention rates alone. Censoring‐weighted and retained‐participant sensitivity analyses yielded similar estimates, but cannot exclude bias from unmeasured determinants of follow‐up. Interpretation of the censoring‐weighted estimates also depends on adequate specification of the censoring model and sufficient probability of remaining observed. Month‐36 remission estimates therefore warrant particular caution.

All treated participants received SLIT; these data therefore do not support comparisons between sublingual and subcutaneous routes. Although written dosing logs and vial preparation and delivery records were used to monitor adherence and continuity, these sources do not directly establish ingestion of every prescribed dose. No cohort‐wide quantitative adherence estimate is presented. Unmeasured differences in actual exposure and treatment persistence may have influenced the observed associations. The 36‐month treatment duration describes the intended course rather than verified completion in all initiators.

FeNO provided an inflammatory correlate, but no cellular or molecular measurements were available to establish mechanism. Prospective studies and pragmatic randomized trials should use harmonized definitions of allergic respiratory disease progression and remission, incorporate mechanistic biomarkers, characterize actual treatment exposure, and determine whether favorable trajectories persist after completion of immunotherapy.

### 5.1 Software and Reproducibility

Data management, propensity‐score estimation, weighting, regression, sensitivity analyses, balance diagnostics, and visualization were implemented in Python using reproducible analytical workflows.

## Author Contributions

**Víctor Gonzalez‐Uribe:** conceptualization, investigation, funding acquisition, writing – original draft, writing – review and editing, validation, methodology, formal analysis, project administration, supervision, data curation. **Sebastian Moreno‐Castro:** investigation, writing – review and editing, visualization, formal analysis. **Viviana Cardoso‐Perez:** investigation, visualization, methodology, formal analysis. **Diego A Mares‐Mateos:** investigation, methodology, writing – review and editing, data curation. **Mariel Paez‐Reyes:** data curation, writing – review and editing, methodology, investigation. **Jimena Prieto‐Gomez:** investigation, validation, formal analysis. **Ricardo Martinez‐Tenopala:** investigation, validation, formal analysis. **Paola de Baro Alvarez:** supervision, data curation, formal analysis, writing – review and editing, writing – original draft, investigation.

## Ethics Statement

The Research Committee of Universidad La Salle, Mexico, the authorized institutional body, determined that this retrospective observational analysis of routinely collected, de‐identified clinical data was exempt from formal ethics review. No study‐specific intervention or procedure was performed.

## Consent

The authorized institutional body determined that informed consent was not required for this retrospective analysis of routinely collected, de‐identified clinical data.

## Conflicts of Interest

Víctor Gonzalez‐Uribe has received honoraria, consulting and/or advisory fees from AbbVie, Sanofi, AstraZeneca, GSK, Faes Farma, ALK‐DIEMSA, Opella, and Bayer. The remaining authors declare no conflicts of interest.

## Permission to Reproduce Material

Not applicable. No material from other sources has been reproduced.

## Clinical Trial Registration

Not applicable. This was a retrospective observational study, and no intervention was assigned by the investigators.

## Supporting information


Supporting File 1

Supporting File 2

## Data Availability

The de‐identified data that support the findings are available from the corresponding author upon reasonable request, subject to institutional approval and applicable data‐protection requirements.
