# Supplementary material for: Allergen Immunotherapy and 36‐Month Allergic Respiratory Disease Trajectories: A Target‐Trial
Source: Immun Inflamm Dis. 2026 Oct 26;14(10):e70569. doi: 10.1002/iid3.70569 (PMC13710663; doi:10.1002/iid3.70569)
Supplement: Supplementary file 1 [file IID3-14-e70569-s001.docx]

**Supporting Information**

**Allergen immunotherapy and 36-month allergic respiratory disease trajectories: a target-trial**

*Víctor Gonzalez-Uribe, Sebastian Moreno-Castro, Viviana Cardoso-Perez, Diego A. Mares-Mateos, Mariel Paez-Reyes, Jimena Prieto-Gomez, Ricardo Martinez-Tenopala, and Paola de Baro Alvarez*

## Table S1. Target-trial specification

| **Component** | **Hypothetical target trial** | **Observational emulation** |
| --- | --- | --- |
| Eligibility | AIT-eligible allergic respiratory disease | Physician-diagnosed allergic rhinitis, asthma, or both with clinically relevant sensitization and baseline AIT eligibility; all index dates allowed a potential 36-month follow-up by study closure |
| Treatment strategies | Initiate AIT within 14 days vs do not initiate AIT | Observed SLIT initiation within 0-14 days vs eligible non-initiation; planned SLIT duration 36 months |
| Time zero | Eligibility and treatment-decision assessment | Index date; identical definition in both groups |
| Grace period | 14 days | Median AIT start 7 days after time zero (IQR 3-11; range 0-14) |
| Assignment | Random | Observed; confounding addressed using baseline propensity-score overlap weighting |
| Follow-up | 36 months | Time zero through 36 months or last available follow-up; inclusion and follow-up within 2020-2025 at AlergiaMx, Mexico City |
| Primary estimand | Effect of assignment to initiation strategy | Treatment-policy estimand approximating intention-to-treat |
| Primary outcome | Clinically meaningful disease worsening | Prespecified phenotype-specific composite |
| Secondary outcomes | Remission, treatment de-escalation, longitudinal control/inflammation | Same |

AIT, allergen immunotherapy; IQR, interquartile range. SLIT, sublingual immunotherapy.

## Table S2. Prespecified operational definitions

| **Outcome** | **Operational definition** |
| --- | --- |
| Clinically meaningful disease worsening | Baseline asthma: exacerbation, sustained GINA escalation, persistent ACT/ACQ deterioration, or sustained increase in OCS burden; allergic rhinitis without baseline asthma: incident physician-diagnosed asthma |
| Sustained disease progression | Primary progression construct excluding isolated exacerbations |
| Clinical remission | At 36 months: controlled disease plus no exacerbations and no OCS bursts during months 24-36 |
| Treatment-associated remission | Clinical remission plus at least one GINA-step reduction or maintenance at minimum clinically appropriate intensity |
| GINA step-down | Reduction of at least one treatment step from baseline |
| Exacerbation rate | Number of exacerbations per annual follow-up interval |
| OCS-burst rate | Number of systemic corticosteroid courses per annual follow-up interval |

*ACQ, Asthma Control Questionnaire; ACT, Asthma Control Test; GINA, Global Initiative for Asthma; OCS, oral corticosteroid.*

## Table S3. Baseline characteristics before and after overlap weighting

| **Characteristic** | **AIT** | **Untreated** | **AIT (OW)** | **Untreated (OW)** | **SMD pre** | **SMD post** |
| --- | --- | --- | --- | --- | --- | --- |
| Age, years | 20.5 (13.0) | 19.6 (12.7) | 19.9 | 19.9 | 0.071 | 0.000 |
| Female sex | 1,241 (63.0) | 464 (62.5) | 62.8% | 62.8% | 0.009 | 0.001 |
| Diagnosis: AR | 307 (15.6) | 121 (16.3) | 16.2% | 16.2% | 0.020 | 0.000 |
| Diagnosis: asthma | 641 (32.5) | 220 (29.6) | 30.3% | 30.3% | 0.062 | 0.000 |
| Diagnosis: asthma + AR | 938 (47.6) | 357 (48.1) | 48.2% | 48.2% | 0.010 | 0.000 |
| Diagnosis: asthma + AR + AD | 85 (4.3) | 44 (5.9) | 5.4% | 5.4% | 0.073 | 0.000 |
| Severity: mild | 1,281 (65.0) | 542 (73.0) | 71.0% | 71.1% | 0.175 | 0.001 |
| Severity: moderate | 510 (25.9) | 143 (19.3) | 20.9% | 20.8% | 0.158 | 0.001 |
| Severity: severe | 180 (9.1) | 57 (7.7) | 8.1% | 8.1% | 0.052 | 0.000 |
| Sensitized allergen groups, n | 3.4 (1.3) | 3.5 (1.3) | 3.4 | 3.4 | 0.053 | 0.000 |
| Single-group sensitization | 126 (6.4) | 42 (5.7) | 5.8% | 5.8% | 0.031 | 0.001 |
| Blood eosinophils, cells/µL | 283 (189) | 278 (176) | 279 | 279 | 0.025 | 0.000 |
| Total IgE, kU/L | 284 (258) | 305 (319) | 298 | 298 | 0.072 | 0.000 |
| FeNO, ppb | 35.3 (18.5) | 34.5 (17.9) | 34.7 | 34.7 | 0.044 | 0.000 |
| Quality of life, 1-7 | 4.82 (0.99) | 4.90 (0.95) | 4.88 | 4.88 | 0.081 | 0.001 |
| Exacerbations, previous 12 months | 0.50 (0.80) | 0.42 (0.79) | 0.44 | 0.44 | 0.097 | 0.000 |
| OCS bursts, previous 12 months | 0.39 (0.69) | 0.32 (0.65) | 0.34 | 0.34 | 0.106 | 0.000 |

*Unweighted sample sizes were AIT n=1,971 and untreated n=742. Values are mean (SD) or n (%) before weighting and weighted mean or proportion after overlap weighting. AD, atopic dermatitis; AIT, allergen immunotherapy; AR, allergic rhinitis; FeNO, fractional exhaled nitric oxide; IgE, immunoglobulin E; OCS, oral corticosteroid; OW, overlap weighted; SMD, standardized mean difference. Sensitization count is the number of distinct allergen groups, not individual aeroallergens; multiple positive tree pollens count as one group. Mono- and polysensitization denote one and more than one sensitized group, respectively.*

## Table S4. Components and burden of disease worsening

| **Outcome** | **AIT** | **Untreated** | **RR (95% CI)** |
| --- | --- | --- | --- |
| Clinically meaningful disease worsening | 46.3% | 63.6% | 0.73 (0.65-0.82) |
| Any exacerbation* | 53.0% | 71.9% | 0.74 (0.69-0.79) |
| Emergency department visit* | 15.0% | 24.3% | 0.62 (0.52-0.74) |
| Hospitalization* | 4.7% | 8.1% | 0.58 (0.41-0.82) |
| Sustained increase in OCS burden* | 7.0% | 15.3% | 0.46 (0.35-0.59) |
| Sustained GINA escalation* | 0.9% | 1.4% | 0.60 (0.26-1.38) |
| Persistent loss of asthma control* | 0.5% | 2.0% | 0.23 (0.09-0.58) |
| Incident asthma† | 9.2% | 14.0% | 0.66 (0.37-1.16) |
| Sustained disease progression‡ | 8.4% | 17.0% | 0.49 (0.35-0.69) |

**Among patients with asthma at baseline (AIT, n=1,664; untreated, n=621). †Among patients with allergic rhinitis without asthma at baseline. ‡Excludes isolated exacerbations. Values are overlap-weighted proportions. RRs and 95% CIs were estimated using overlap-weighted modified Poisson regression with robust standard errors. AIT, allergen immunotherapy; CI, confidence interval; GINA, Global Initiative for Asthma; OCS, oral corticosteroid; RR, relative risk.*

## Table S5. Follow-up retention and censoring sensitivity

| **Follow-up** | **AIT available** | **Untreated available** |
| --- | --- | --- |
| 2 months | 97.4% | 92.9% |
| 6 months | 92.8% | 88.3% |
| 12 months | 87.6% | 82.7% |
| 18 months | 80.6% | 75.7% |
| 24 months | 74.6% | 70.5% |
| 36 months | 65.2% | 57.4% |

*At 36 months, 1,712/2,713 participants (63.1%) remained evaluable. IPCW-adjusted estimates were RR 2.59 (95% CI 1.94-3.45) for clinical remission and RR 5.64 (95% CI 3.32-9.59) for treatment-associated remission. AIT, allergen immunotherapy; CI, confidence interval; IPCW, inverse probability-of-censoring weighting; RR, relative risk.*

## Table S6. Sensitivity analyses

| **Analysis** | **n** | **AIT** | **Untreated** | **RR (95% CI)** |
| --- | --- | --- | --- | --- |
| Primary overlap weighting | 2,713 | 46.3% | 63.6% | 0.73 (0.65-0.82) |
| Sustained progression | 2,713 | 8.4% | 17.0% | 0.49 (0.35-0.69) |
| Expanded propensity-score model | 2,713 | 46.4% | 63.6% | 0.73 (0.65-0.82) |
| Stabilized IPTW, 1st-99th truncation | 2,713 | 47.5% | 64.7% | 0.73 (0.68-0.79) |
| Restricted common support | 2,629 | 46.4% | 63.4% | 0.73 (0.65-0.82) |
| Asthma at baseline only | 2,285 | 53.5% | 73.1% | 0.73 (0.66-0.81) |
| 36-month retained only | 1,712 | 46.5% | 64.8% | 0.72 (0.62-0.83) |

*Values are weighted proportions unless otherwise indicated. AIT, allergen immunotherapy; CI, confidence interval; IPTW, inverse probability of treatment weighting; RR, relative risk.*

## Table S7. Effect heterogeneity

| **Subgroup** | **AIT risk** | **Untreated risk** | **RR (95% CI)** | **p interaction** |
| --- | --- | --- | --- | --- |
| Adult | 43.0% | 63.0% | 0.68 (0.58-0.80) | 0.244 (age) |
| Pediatric | 50.1% | 64.2% | 0.78 (0.67-0.91) |  |
| Female | 47.7% | 65.0% | 0.73 (0.64-0.84) | 0.894 (sex) |
| Male | 44.1% | 61.1% | 0.72 (0.60-0.87) |  |
| Mild disease | 37.0% | 54.7% | 0.68 (0.58-0.79) | 0.049 (severity) |
| Moderate disease | 63.8% | 82.5% | 0.77 (0.66-0.91) |  |
| Severe disease | 83.3% | 93.0% | 0.90 (0.77-1.05) |  |
| One sensitized group | 43.6% | 55.6% | 0.78 (0.47-1.31) | 0.770 |
| Multiple sensitized groups | 46.5% | 64.1% | 0.73 (0.65-0.81) |  |
| 1-2 sensitized groups | 45.1% | 58.2% | 0.78 (0.61-0.99) | 0.668 |
| 3-4 sensitized groups | 47.5% | 64.5% | 0.74 (0.63-0.85) |  |
| ≥5 sensitized groups | 44.8% | 67.2% | 0.67 (0.52-0.85) |  |

*Subgroup analyses were exploratory. Heterogeneity was evaluated with treatment-by-subgroup interaction terms; no multiplicity adjustment was applied. AIT, allergen immunotherapy; CI, confidence interval; RR, relative risk. Sensitization subgroups are defined at the allergen-group level; multiple positive tree pollens count as one group.*

## Table S8. Longitudinal clinical and inflammatory outcomes

| **Outcome** | **12 months** | **24 months** | **36 months** |
| --- | --- | --- | --- |
| ACT, adjusted difference | +1.34 (1.19-1.48) | +1.91 (1.74-2.08) | +2.12 (1.93-2.31) |
| ACQ, adjusted difference | -0.28 (-0.30 to -0.25) | -0.40 (-0.43 to -0.37) | -0.48 (-0.51 to -0.44) |
| Quality of life, adjusted difference | +0.40 (0.36-0.43) | +0.60 (0.56-0.64) | +0.69 (0.64-0.74) |
| FeNO, adjusted difference (ppb) | -4.93 (-5.44 to -4.42) | -6.92 (-7.58 to -6.26) | -8.03 (-8.73 to -7.32) |

*Adjusted between-group differences and 95% confidence intervals were estimated from overlap-weighted longitudinal models incorporating the baseline outcome and follow-up time. ACT, Asthma Control Test; ACQ, Asthma Control Questionnaire; FeNO, fractional exhaled nitric oxide.*

*Time zero was the common eligibility and treatment-decision assessment (day 0). The 14-day grace period allowed routine clinical initiation of allergen immunotherapy. Participants were classified by initiation versus eligible non-initiation and followed for up to 36 months or until the last available assessment.*

# Table S9. Detected aeroallergen sensitization

Percentages refer to the full eligible cohort (N=2,713).

| **Detected sensitization** | **Percentage** |
| --- | --- |
| House dust mites | 62.8% |
| Cynodon dactylon | 48.9% |
| Phleum pratense | 18.9% |
| Fraxinus excelsior | 52.2% |
| Quercus | 35.6% |
| Cypress | 48.9% |
| Rumex | 25.0% |
| Ambrosia | 22.2% |
| Artemisia | 17.2% |
| Plantago | 9.4% |
| Cat | 31.1% |
| Dog | 27.8% |

Categories are not mutually exclusive because patients may be sensitized to more than one aeroallergen. These values describe detected sensitization and not the composition of immunotherapy formulations. These frequencies describe the listed aeroallergens, whereas the count in Table S3 refers to allergen groups. A patient positive to Fraxinus, Quercus and cypress contributes to each listed frequency but only one tree-pollen group to the baseline count; the percentages therefore need not sum to 100% or reproduce the group-count mean.

Among participants sensitized to a single allergen group (n=168; AIT, n=126; untreated, n=42), 23% were sensitized to house dust mites, 45% to trees, and 32% to other allergens. Percentages are descriptive and unweighted. Exact counts by individual allergen are not presented.

# Table S10. SLIT treatment characteristics

Distribution of allergens used among SLIT initiators (n=1,971).

| **Allergen or formulation characteristic** | **Percentage of treated patients** |
| --- | --- |
| House dust mites | 65.6% |
| Cupressus spp. | 62.3% |
| Fraxinus excelsior | 48.7% |
| Quercus spp. | 33.7% |
| Cynodon dactylon | 24.0% |
| Other aeroallergens | 52.6% |

Categories are not mutually exclusive because formulations may contain more than one allergen. Percentages are descriptive and unweighted and use treated patients, rather than the full cohort, as the denominator.

## Route and planned duration

All AIT initiators received sublingual immunotherapy. The planned treatment duration was 36 months. This does not imply verified treatment completion or an available month-36 assessment for every initiator.

## Monitoring adherence and continuity

Patients maintained written dosing calendars or administration logs. AlergiaMx also recorded preparation of new immunotherapy vials and their delivery dates. These sources were used together to verify reported dosing and treatment continuity for individual patients. No cohort-wide quantitative adherence rate is reported.
