# Supplementary material for: Allergen Immunotherapy and 36‐Month Allergic Respiratory Disease Trajectories: A Target‐Trial
Source: Immun Inflamm Dis. 2026 Oct 26;14(10):e70569. doi: 10.1002/iid3.70569 (PMC13710663; doi:10.1002/iid3.70569)
Supplement: Supplementary file 2 [file IID3-14-e70569-s002.docx]

STROBE checklist for cohort studies

**Allergen immunotherapy and 36-month allergic respiratory disease trajectories: a target-trial**

Víctor Gonzalez-Uribe and colleagues | Immunity, Inflammation and Disease

Design: retrospective observational cohort study using target-trial emulation. This checklist maps all 22 STROBE items and their subitems to the supplied manuscript and supporting information. Entries distinguish reported information from reporting gaps.

M = main manuscript (12 pages); S = Supporting Information (11 pages). Page references use the revised files as rendered for this review, counting each title page as page 1. Section and table names identify the location if pagination changes. “Not reported” is not equivalent to “Not applicable”.

| **Item** | **STROBE recommendation** | **Page location and reporting details** |
| --- | --- | --- |
| **1a** | (a) Indicate the study’s design with a commonly used term in the title or the abstract | Reported. M p. 2, Abstract, Materials and methods: explicitly identifies a single-center retrospective cohort using target-trial emulation. |
| **1b** | (b) Provide in the abstract an informative and balanced summary of what was done and what was found | **Reported.** M p. 2, Abstract. Summarizes the comparison, 36-month follow-up, overlap weighting, sample size, principal estimates with confidence intervals, and an association-based conclusion. |
| **Introduction** | | |
| **2** | Explain the scientific background and rationale for the investigation being reported | **Reported.** M p. 3, Introduction, paragraphs 1–4. Rationale concerns allergic disease trajectories, potential disease modification, and limitations of routine-care comparisons. |
| **3** | State specific objectives, including any prespecified hypotheses | **Reported.** M p. 3, final Introduction paragraph. States the comparison and hypotheses concerning worsening, remission, treatment de-escalation, and clinical and inflammatory trajectories. |
| **Methods** | | |
| **4** | Present key elements of study design early in the paper | **Reported.** M p. 3, Study design and target-trial framework; S p. 1, Table S1. Retrospective longitudinal observational design, target strategies, time zero, follow-up, and estimand are described. |
| **5** | Describe the setting, locations, and relevant dates, including periods of recruitment, exposure, follow-up, and data collection | Reported for setting and observation period. M pp. 3–4, Study design; S p. 1, Table S1. AlergiaMx, Mexico City; inclusion and follow-up during 2020–2025; index dates permitted potential 36-month follow-up. Separate extraction dates and exact first/last index dates are not specified. |
| **6a** | (a) Give the eligibility criteria, and the sources and methods of selection of participants. Describe methods of follow-up | **Partial.** M pp. 4–5, Study population, Treatment strategies and time zero, and Missing data, sensitivity, and subgroup analyses; S pp. 1 and 5, Tables S1 and S5. Eligibility and follow-up visits are described; the method of selecting records from the source population is not specified. |
| **6b** | (b) For matched studies, give matching criteria and number of exposed and unexposed | **Not applicable.** No matched design was used. The treatment groups were compared using propensity-score overlap weighting (M p. 5). |
| **7** | Clearly define all outcomes, exposures, predictors, potential confounders, and effect modifiers. Give diagnostic criteria, if applicable | Partial. M pp. 4–6, Treatment strategies, Baseline characteristics, Outcomes, Sustained progression, and Subgroup analyses; S pp. 1–2, Tables S1–S2. Main variables and outcome thresholds are defined. Diagnostic criteria, severity classification, and the operational definition of an exacerbation are not fully specified. |
| **8** | For each variable of interest, give sources of data and details of methods of assessment (measurement). Describe comparability of assessment methods if there is more than one group | **Partial.** M pp. 3–5, Methods; S pp. 2–3, Tables S2–S3. Routinely collected clinical data and named outcomes are described. The quality-of-life instrument, age-specific instrument use, measurement protocols, and comparability of ascertainment between groups are not detailed. |
| **9** | Describe any efforts to address potential sources of bias | Reported. M pp. 3–5, target-trial framework, treatment strategies, overlap weighting and sensitivity analyses; M pp. 8–9, Discussion and limitations. Time-zero alignment, eligible comparator, balance checks and censoring adjustment are described. |
| **10** | Explain how the study size was arrived at | Not reported. M p. 6, Study population, and Figure 1 give source and eligible counts (2,835 and 2,713), but do not explain the basis of study size or whether every eligible record was included. |
| **11** | Explain how quantitative variables were handled in the analyses. If applicable, describe which groupings were chosen and why | Partial. M pp. 4–6, Outcomes and Statistical analysis; S pp. 3 and 7, Tables S3 and S7. Outcome thresholds and sensitization categories are given. Figure 2 defines pediatric age as <18; the rationale for categorization and functional forms of continuous covariates are not specified. The revised Methods and Tables S3, S7 and S9 distinguish allergen-group counts from individual aeroallergen frequencies. |
| **12a** | (a) Describe all statistical methods, including those used to control for confounding | Partial. M p. 5, weighting and Statistical analysis; M p. 9, Software. Covariates, weighting equations, regression families and robust uncertainty are reported. Full longitudinal model specification and expanded-model covariates are not provided. |
| **12b** | (b) Describe any methods used to examine subgroups and interactions | Reported. M pp. 5–6, subgroup methods; S p. 7, Table S7. Exploratory interaction tests; no multiplicity adjustment. |
| **12c** | (c) Explain how missing data were addressed | Partial. M pp. 5–6, Missing data; M p. 12, Figure 4 legend; S p. 5, Table S5. Censoring weighting and available repeated measurements are described; missing baseline covariates and intermittent missing outcomes are not explicitly addressed. |
| **12d** | (d) If applicable, explain how loss to follow-up was addressed | Reported. M pp. 5–7, Missing data and Robustness; M p. 9, limitations; S pp. 5–6, Tables S5–S6. Censoring weights and retained-participant sensitivity analysis; residual selection bias acknowledged. |
| **12e** | (e) Describe any sensitivity analyses | Reported. M pp. 5–7, Methods and Robustness; S pp. 5–6, Tables S5–S6. Alternative endpoints, models, weighting and population restrictions. |
| **Results** | | |
| **13a** | (a) Report numbers of individuals at each stage of study—eg numbers potentially eligible, examined for eligibility, confirmed eligible, included in the study, completing follow-up, and analysed | Partial. M p. 6, Results; M p. 12, Figure 3 legend; Figure 1; S pp. 5–6. Reports source, eligible, treated, baseline-asthma and total month-36 counts. Exact group-specific remission denominators and other outcome-specific analysis counts are not reported. |
| **13b** | (b) Give reasons for non-participation at each stage | **Partial.** M p. 4, Treatment strategies and time zero, lists reasons for AIT non-initiation. Figure 1 reports 122 exclusions, but their reasons and counts by reason are absent. Reasons for loss to follow-up are not provided. |
| **13c** | (c) Consider use of a flow diagram | Reported. Figure 1 and legend, M p. 12. Source cohort, exclusions, eligible groups and baseline-asthma counts. |
| **14a** | (a) Give characteristics of study participants (eg demographic, clinical, social) and information on exposures and potential confounders | Reported. S p. 3, Table S3; M pp. 4 and 6, Baseline characteristics and Results. Group-specific baseline characteristics and weighting; S pp. 10–11, Tables S9–S10, sensitization and SLIT characteristics. |
| **14b** | (b) Indicate number of participants with missing data for each variable of interest | **Not reported.** Per-variable missing-data counts, separately by treatment group, are not provided. S p. 5, Table S5 reports visit availability percentages, which do not identify missingness for individual variables. |
| **14c** | (c) Summarise follow-up time (eg, average and total amount) | Partial. M pp. 3–6; S pp. 1 and 5, Tables S1 and S5. Potential versus observed follow-up and visit retention are distinguished. Mean/median observed follow-up and total person-time are not reported. |
| **15** | Report numbers of outcome events or summary measures over time | Partial. M pp. 6–7 and p. 12, Table 1; S pp. 4 and 8, Tables S4 and S8; Figures 3–4. Weighted risks and longitudinal summaries are reported; raw event counts and outcome-specific denominators are not supplied. |
| **16a** | (a) Give unadjusted estimates and, if applicable, confounder-adjusted estimates and their precision (eg, 95% confidence interval). Make clear which confounders were adjusted for and why they were included | Partial. M pp. 4–7 and p. 12, Table 1; S pp. 4 and 8. Adjusted estimates, precision, covariates and rationale for confounder selection are reported. Unadjusted effect estimates are not reported. |
| **16b** | (b) Report category boundaries when continuous variables were categorized | Partial. S p. 7, Table S7, gives sensitized allergen-group categories (1–2, 3–4, ≥5); M pp. 4–5 gives outcome thresholds. Figure 2 identifies pediatric age as <18; methods do not explicitly define this boundary or the rationale for categorization. |
| **16c** | (c) If relevant, consider translating estimates of relative risk into absolute risk for a meaningful time period | Reported. M pp. 2, 6 and 12, Table 1; S p. 4, Table S4. Absolute weighted risks accompany relative risks over the specified observation period. |
| **17** | Report other analyses done—eg analyses of subgroups and interactions, and sensitivity analyses | Reported. M p. 7, Robustness and heterogeneity; S pp. 5–8, Tables S5–S8. Sensitivity, subgroup and longitudinal analyses; numerical diagnostic-phenotype subgroup estimates are not tabulated. |
| **Discussion** | | |
| **18** | Summarise key results with reference to study objectives | Reported. M p. 7, opening Discussion paragraphs. Main associations are summarized in relation to the objectives. |
| **19** | Discuss limitations of the study, taking into account sources of potential bias or imprecision. Discuss both direction and magnitude of any potential bias | Reported. M p. 9, limitations. Residual confounding, differential follow-up and exposure uncertainty; direction and magnitude of selection bias cannot be established from observed retention alone. Sensitivity analyses do not eliminate unmeasured bias. |
| **20** | Give a cautious overall interpretation of results considering objectives, limitations, multiplicity of analyses, results from similar studies, and other relevant evidence | Reported. M pp. 7–9, Discussion and limitations; S p. 7, Table S7. Cautious noncausal interpretation, exploratory heterogeneity, REACT/TARGET/GAP context and distinction from off-treatment remission. |
| **21** | Discuss the generalisability (external validity) of the study results | Partial. M pp. 8–9, Discussion and limitations. Routine-practice heterogeneity and restriction to SLIT are addressed; broader generalisability to other settings is not explicitly developed. |
| **Other information** | | |
| **22** | Give the source of funding and the role of the funders for the present study and, if applicable, for the original study on which the present article is based | Reported. M p. 10, Acknowledgements and funding. No external funding. Conflict disclosures also appear on p. 10. |

Items 8, 13, 14, and 15 call for information separately for exposed and unexposed groups. A completed checklist documents reporting; it does not certify that every recommendation has been fully met.

Source: STROBE Statement, cohort-study checklist supplied through the EQUATOR Network. Recommendation wording retained from the official form; location and reporting-detail column completed for this manuscript.
https://www.equator-network.org/reporting-guidelines/strobe/
von Elm E, et al. PLoS Med. 2007;4(10):e296. doi:10.1371/journal.pmed.0040296.
